# Diagnosis Methodology Based on Deep Feature Learning for Fault Identification in Metallic, Hybrid and Ceramic Bearings

**DOI:** 10.3390/s21175832

**Published:** 2021-08-30

**Authors:** Juan Jose Saucedo-Dorantes, Francisco Arellano-Espitia, Miguel Delgado-Prieto, Roque Alfredo Osornio-Rios

**Affiliations:** 1 HSPdigital CA-Mecatronica Engineering Faculty, Autonomous University of Queretaro, San Juan del Rio 76806, Mexico; raosornio@hspdigital.org; 2 MCIA Department of Electronic Engineering, Technical University of Catalonia (UPC), 08034 Barcelona, Spain; francisco.arellano@upc.edu (F.A.-E.); miguel.delgado@upc.edu (M.D.-P.)

**Keywords:** fault diagnosis, bearings, deep learning, autoencoder, vibration signal, multi-domain feature extraction

## Abstract

Scientific and technological advances in the field of rotatory electrical machinery are leading to an increased efficiency in those processes and systems in which they are involved. In addition, the consideration of advanced materials, such as hybrid or ceramic bearings, are of high interest towards high-performance rotary electromechanical actuators. Therefore, most of the diagnosis approaches for bearing fault detection are highly dependent of the bearing technology, commonly focused on the metallic bearings. Although the mechanical principles remain as the basis to analyze the characteristic patterns and effects related to the fault appearance, the quantitative response of the vibration pattern considering different bearing technology varies. In this regard, in this work a novel data-driven diagnosis methodology is proposed based on deep feature learning applied to the diagnosis and identification of bearing faults for different bearing technologies, such as metallic, hybrid and ceramic bearings, in electromechanical systems. The proposed methodology consists of three main stages: first, a deep learning-based model, supported by stacked autoencoder structures, is designed with the ability of self-adapting to the extraction of characteristic fault-related features from different signals that are processed in different domains. Second, in a feature fusion stage, information from different domains is integrated to increase the posterior discrimination capabilities during the condition assessment. Third, the bearing assessment is achieved by a simple softmax layer to compute the final classification results. The achieved results show that the proposed diagnosis methodology based on deep feature learning can be effectively applied to the diagnosis and identification of bearing faults for different bearing technologies, such as metallic, hybrid and ceramic bearings, in electromechanical systems. The proposed methodology is validated in front of two different electromechanical systems and the obtained results validate the adaptability and performance of the proposed approach to be considered as a part of the condition-monitoring strategies where different bearing technologies are involved.

## 1. Introduction

Scientific and technological advances around rotatory electrical machinery are leading to increased efficiency and performance of the processes and systems in which they take part. In this regard, the consideration of advanced materials, such as hybrid or ceramic bearings, are of high interest in the move towards high-performance and oil-free rotatory electromechanical actuators. High demanding operative requirements have become the main characteristics of modern rotating machinery as well as their continuous operation, which also increases the risk of system failure and, specifically, the acceleration of bearing faults as one of the most common sources of malfunction [1,2].

Depending on the considered bearing technology, that is, metallic, hybrid, or full ceramic, the operative characteristics such as friction coefficient, thermal rejection ratio, or lubrication requirement among others differ. The suitability of these features to the application is, in fact, the motivation to decide the incorporation of one or another bearing technology. Metallic bearings are commonly used in simple and common applications of everyday life; the most complex applications for metallic bearings are probably those related to industrial machines, whereas ceramic bearings are being used in particular applications such as in high-speed lathes, chemical machinery and aeroengines, among others. In this regard, in environments in which high-speed, heavy-duty, low noise, and/or high precision are required, full ceramic bearings are being gradually incorporated [3]. However, from the maintenance point of view, different bearing technologies imply several differences in the degradation processes and also in the expected characteristic fault-related patterns that are usually considered in the design process of the condition-based maintenance solutions [4,5,6,7]. Thus, the differences in the fault mechanisms due to differences in brittleness or toughness between steel and ceramic have effects on the vibration response during the machine operation [8,9,10,11,12]. Specifically, overall vibration levels in full-ceramic bearings are modified by the rotational speed and do not suffer changes in presence of axial load. Meanwhile, under the influence of a given axial load and rotational speed, the vibratory levels of full-ceramic bearing are reduced, and for hybrid bearings they increase [13,14]. In this regard, although the mechanical principles from which the characteristic fault-related effects are estimated remain, the quantitative analysis over the vibration signals is affected in amplitude and frequency terms.

Indeed, there is no consensus related to the most convenient signal processing stage to face different bearing technologies. On the other side, concerning the condition assessment and fault identification in different bearing technologies, a great deal of approaches have been proposed and focused on solving the following problems: (i) the occurrence of faults on different parts of the bearing, where, the fault assessment in the inner race, outer race, balls and cage, have been widely studied [3,4,15,16]; (ii) the assessment and recognition of the bearing fault severity which has been normally analyzed over the inner race and outer race [9,12,17]; and (iii) the analysis and prediction of the degradation in any of the bearing elements with the aim of estimating its remaining useful life [6,18]. Despite the fact that several condition-monitoring approaches have been successfully proposed in the field of bearing fault identification to face the most common problems related to the assessment of faulty conditions, most of these proposals have been designed for being applied to a specific bearing technology, the metallic bearings. Under this premise and in order to provide solutions in front of the emerged bearing technologies, the proposal of new condition assessment approaches capable of self-adapting, independently of the bearing technology, such as metallic, hybrid and ceramic bearings, is mandatory. In actuality, it is possible to find works in the related literature of condition monitoring that consider different domains of analysis, that is, time domain, frequency domain and/or time–frequency domain [19,20,21]. Thus, motivated by the consideration that the characteristic fault-related patterns are modulated by the electromechanical system configuration and operating conditions in which the bearing operates, this leads to data-driven approaches as a more suitable solution for practical solutions and applications. In recent years, proposals that have been addressed the fault detection and identification by using artificial intelligence (AI)-based models have attracted great attention [22,23]. Initially, considering machine learning (ML) approaches, such as the work presented by A. Soualhi et al. in [24], based on support vector machines, or the work presented by J. J. Saucedo et al. in [25], based on artificial neural networks (NN), or even the work presented by J. Guo et al. in [26], based on *k*-nearest neighbors. However, despite the fault detection and identification can be carried out by such classical approaches in which depending on the bearing technology and its electromechanical context, the most significant fault-related features have to be selected, and recently, deep learning (DL) approaches are being considered. The use of DL has attracted attention since DL provides an adaptive framework that allows the design of architecture that can be adapted in the solution of specific problems that must be faced in which the engineering features and AI algorithms coexist. Hence, DL-based approaches have recently been established as a powerful tool to adapt the pattern characterization of electromechanical systems [27]. Significant examples of this fact are the works presented by J. Dai et al., X. J. Gou et al. or G. B. Huang et al. in [15,22,28], respectively.

However, dealing with the generalization of the diagnosis methodology, and specifically, considering its application to different bearing technologies, such as metallic, hybrid and full ceramic, new DL-based procedures have to overcome a set of critical challenges that are avoiding its practical application [29]. Mainly, these limitations are: (i) the operation with limited amounts of data, which are difficult to obtain in industrial applications especially concerning the fault patterns, (ii) understandable interpretation of the learning process, that is not clear for practitioners, (iii) generalization-based approaches avoiding over-fitted solutions and (iv) the methodological hyperparameter tuning procedures to promote ease of access and configuration.

Thereby, the contribution of this work lies in the proposal of a novel data-driven monitoring methodology based on deep learning applied to the diagnosis and identification of bearing faults for different bearing technologies, such as metallic, hybrid and ceramic bearings, in electromechanical systems. Thus, the proposed diagnosis methodology promotes a common framework which valid for different bearing technologies and electromechanical systems. To address this issue, the proposed methodology consists of three main stages. First, a DL-based model is designed with the ability of self-adapting to the extraction of characteristic fault-related features from different signals including classical vibration and stator current; also, the proposed diagnosis approach is designed for being adapted to any other different available physical magnitude. In order to perform the proposed deep feature learning, a distributed DL-based structure supported by a stacked au-to encoder (SAE) is considered for extracting potential and meaningful fault-related features from the available signals that are characterized through its analysis in three different domains (time domain, frequency domain, and time–frequency domain). In this sense, the proposed methodology offers a practical way to implement a DL approach independently of the electromechanical configuration in general, but considering the bearing technology in particular. Second, a feature fusion stage that integrates information from different domains is considered to increase the posterior discrimination capabilities during the condition assessment. Finally, third, a simple softmax layer is included to compute the final classification result. The proposed methodology is validated in front of two different electromechanical systems. First, a self-designed experimental test bench including metallic, hybrid and full ceramic bearings, at multiple operating conditions under the same fault is considered to evaluate the adaptation capabilities of the proposed methodology in front of the bearing technology. Second, a standard benchmark as the Case Western Reserve University bearing data center [30,31], is also evaluated in order to assess the performance of the proposed diagnostic method in front of different metallic bearing failures in a reference framework. It should be noted that the proposed approach is of significant importance in the field, even more so when designing a fault detection and identification solution, since depending on the bearing technology, the electromechanical system configuration, and even the range of operating conditions, the meaning and correlation of characteristic fault-related features will differ. However, the proposed methodology represents a common framework for the design and implementation of feasible solutions to be applied in a wide range of electromechanical systems where the bearings are involved in multiple configurations.

The proposed work is organized as follows: Section 2 presents the theoretical background on the stacked auto-encoder as a deep-learning approach. Section 3 presents the proposed method presented in this study. In Section 4, the results and validation of this proposal are discussed. Finally, the conclusions are provided in Section 5.

## 2. Autoencoder-Based Deep Feature Learning

**Figure 1:**
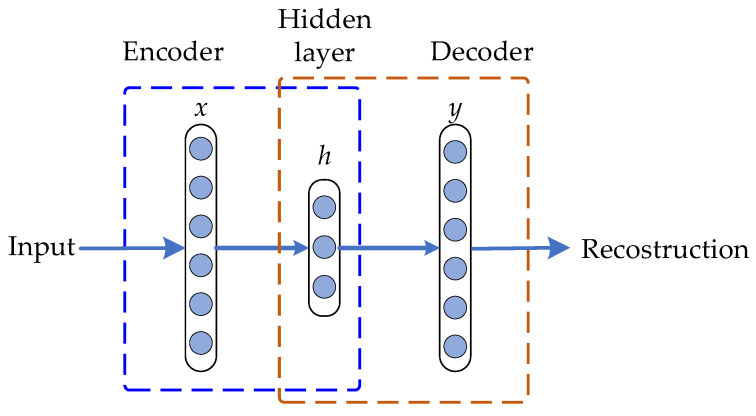
Graphical representation of the structure of a single-layer autoencoder.

Unsupervised feature-learning techniques have been included as a fundamental part in most of the proposed condition-monitoring and diagnosis approaches. In this regard, the autoencoder (AE), which is well-known as an unsupervised NN, has a symmetrical structure and one of its main design purposes is focused to perform the representation of a high-dimensional feature space into a low-dimensional space; specifically, the AE goal is to reconstruct the input pattern at the output of the network by means of minimizing the reconstruction error between the input and output feature patterns [26,32]. The AE network structure is represented by a single hidden layer and the graphic representation is shown in Figure 1. The mathematical notation that belongs to a single AE network structure is described below; however, it should be clarified that aiming to facilitate the description of the mathematical basis, italic letters are used to define scalars, bold-face lower-case letters are considered in the definition of vectors and bold-face upper-case letters are used in the definition of matrices.

Since AE is an unsupervised NN, the training procedure is divided into two main phases: encoder and decoder; thus, during the encoder procedure, an input feature dataset vector is connected to the input encoder layer **x**. The encoder procedure aims to perform the mapping of the input **x** into a hidden representation which is denoted by the hidden layer or encoder vector **h**. Therefore, the hidden layer **h** contains the numerical information and maps that lead to achieve the connection of the input layer **x** with the hidden layer **h** through *n* sparse-activated neurons and non-linear transformation following Equation (1):

(1) $$h=f\left(W_{e}x+B_{e}\right)$$

where f is the sigmoid function used as the non-linear activation function, and $W_{e}$ and $B_{e}$ are the corresponding weights and biases matrices for the encoder phase, respectively. Otherwise, the decoder procedure which is applied as a reverse transformation procedure aims to perform the reconstruction of the input feature vector **x** from the information contained in the hidden layer **h**; thus, an output decoder layer vector **y** is obtained during the decoder procedure, which is achieved by following Equation (2):

(2) $$y=f\left(W_{d}h+B_{d}\right)$$

From Equation (2), the corresponding weights and bias matrices considered in the decoder procedure are $W_{d}$ and $B_{d}$, respectively; the AE output is represented by **y** as the achieved reconstruction of the corresponding input **x**. Therefore, the aim of the AE feature learning process lies in the optimization of the parameters $\theta =\left\{W_{d},B_{e},\;W_{d},\;B_{d}\right\}$, where the minimization of the reconstruction error between the input and the output feature vectors, **x** and **y**, respectively, is accomplished in terms of the mean squared error (MSE) by following Equation (3):

(3) $$MSE=\frac{1}{N}\overset{M}{\underset{k=1}{\sum }}{\left(x_{k}-y_{k}\right)}^{2}$$

where *k* = 1, 2,… *M*, $x_{k}$, $y_{k}$, represents each corresponding element of the input and the out feature vectors, respectively, and *M* is the total number of input and output elements.

Moreover, in order to prevent overfitted responses on AE networks, it is necessary to introduce *L_2_* as the regularization term to the cost or fitness function. Specifically, *L_2_* is denominated as the weight decay regularization term, which is added to avoid the emergence of large weights in the weight matrix W, thus, such a term is formulated as Equation (4) depicts:

(4) $$\Omega_{weights}=\frac{1}{2}\overset{a}{\underset{i=1}{\sum }}\overset{b}{\underset{j=1}{\sum }}\overset{c}{\underset{k=1}{\sum }}{\left(W_{jk}^{i}\right)}^{2}$$

where a is the number of weight parameters, b is the number of rows and c is the number of columns in each weight matrix W, and $W_{jk}^{i}$ represents each element of W.

Furthermore, a sparsity regularizer term is introduced to generate more specific AE network models capable of learning information from the input more effectively. This regularizer term is a function of the average output activation value of a neuron which is described as followed by Equation (5):

(5) $$\hat{\rho_{i}}=\frac{1}{n}\overset{n}{\underset{j=1}{\sum }}f\left(w_{i}^{T}x_{j}+b_{i}\right)$$

where n is the total number of training samples, $x_{j}$ is the $j^{th}$ training sample, $w_{i}^{T}$ is the $i^{th}$ row of the weight matrix $W_{e}$ and $b_{i}$ is the *i*-th entry of the bias vector, $b_{e}$. This purpose of the sparsity function is to restrict the values of $\hat{\rho_{i}}$ to be low, which causes the AE to generate a mapping such that each neuron in the hidden layer is activated with a small number of samples. In this regard, a term $\Omega_{Sparsity}$ is added to the cost function to constrain the $\hat{\rho_{i}}$ values and make the neurons sparce. For this, the *KL* divergence is used to measure the distance between ρ and $\hat{\rho_{i}}$, where ρ is the wanted sparsity parameter and $\hat{\rho_{i}}$ is the effective sparsity of a corresponding hidden neuron

(6) $$\Omega_{Sparsity}=\overset{n}{\underset{i=1}{\sum }}KL(\rho ||\hat{\rho_{i}})=\overset{n}{\underset{i=1}{\sum }}\rho \;log\left(\frac{\rho }{\hat{\rho_{i}}}\right)+\left(1-\rho \right)log\left(\frac{1-\rho }{1-\hat{\rho_{i}}}\right)$$

Subsequently, the fitness function defined as *FunCost* is described as the sum of the error term regularization penalty terms

(7) $$FunCost=MSE+\lambda *\Omega_{weights}+\beta *\Omega_{Sparsity}$$

where λ is the specific parameter for the *L*_2_ regularization term that takes control over the weight decay and β is the corresponding parameter to the sparsity regularization term.

**Figure 2:**
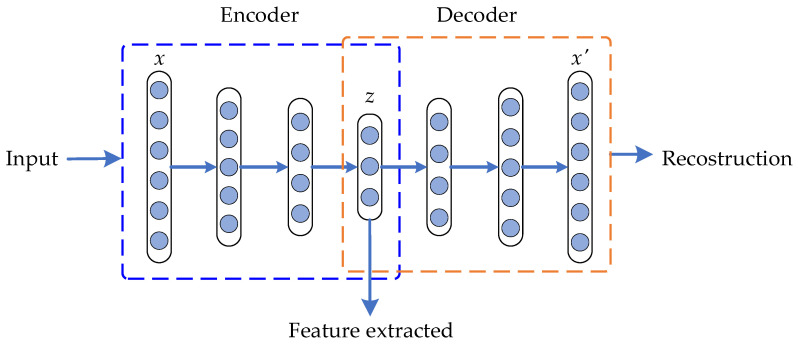
Graphical representation of a stacked AE-based deep neural network structure.

Therefore, the parameter-tuning process can be stated as an optimization problem where the network parameters are adjusted aiming to minimize the resulting cost function [16]. In this paper, the AE network aims to use the ability to learn and reconstruct patterns to characterize the different conditions and operating regimes of rotating systems. In addition, a deep neural network (DNN) can be built from stacking several AEs, as shown in Figure 2. In this way, a DNN based on SAE has a greater capacity to automatically extract fault features through multiple layers and non-linear transformations. The process to efficiently construct a DNN is described by Hilton et al. [33]. The result is a multilayer deep-autoencoder (DAE) structure with a bottleneck coding layer in the middle, which represents the features extracted in a low dimension.

## 3. Deep Feature Learning Based Methodology

**Figure 3:**
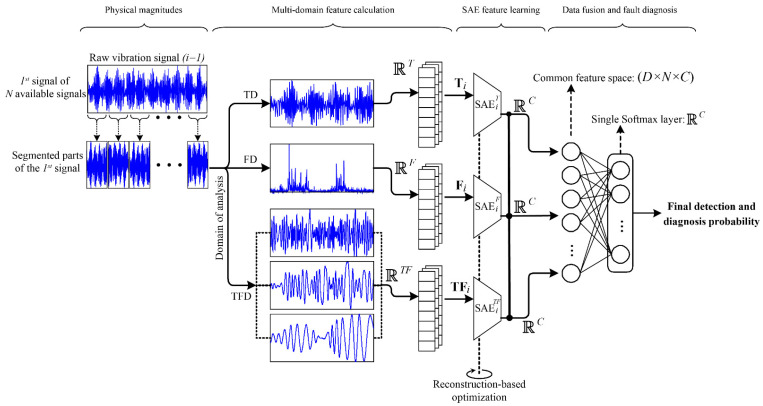
Flow chart of the proposed data-driven diagnosis methodology based on deep feature learning for fault diagnosis applied to the diagnosis and identification of bearing faults for different bearing technologies, such as metallic, hybrid and ceramic bearings, in electromechanical systems.

As mentioned, new condition-monitoring strategies are required for those technological fields in which the demanding requirements of operation over rotatory electromechanical systems are considered. This fact is of special importance dealing with bearings of rotating machines since they are one of the most common sources of malfunctions in related electromechanical systems. Moreover, the bearing technological variants, that is, metallic, hybrid and ceramic, lead to an increased complexity when a common framework for a feasible characterization and posterior pattern recognition is desired. In this regard, the flowchart of the proposed deep feature learning based condition-monitoring methodology focused on the diagnosis and identification of bearing faults for different bearing technologies is shown in Figure 3. The diagnosis methodology has been designed following a proposed step-by-step strategy that facilitates its practical application as a condition-monitoring scheme over electromechanical systems. In this regard, the methodology represents a common framework to face bearing condition characterization and its posterior recognition independently of the bearing’s technology. The proposed methodology includes a SAE-based DNN to perform an automatic characterization of the available acquisitions processed under multiple domains, that is, time, frequency and time–frequency. It must be noted that the general structure of the proposed methodology is intended to serve as a guide to adapt such condition-monitoring procedures to different industrial systems, that is, considering differences in the registering modules (i.e., type and/or number of acquired physical magnitudes), and differences in the electromechanical configuration and operation (i.e., speed, torque, but also the bearing’s technologies).

The consideration of DL techniques as part of the related condition-monitoring methodologies represents a complex task that has not been properly solved by most of the available studies in the field. The selection and configuration of the related hyperparameters as a result of wrapper or empirical approaches leads to a high risk of overfitting during the validation of the methodology. In this regard, the proposed three-stage scheme promotes the generalization of the resulting model by introducing the selection of the hyperparameters as part of the methodology, thus leading to a feasible method easy to implement and adaptable to different electromechanical configurations.

### 3.1. Multi-Domain Feature Calculation

Indeed, the proposed methodology has been designed for being adapted to different and/or multiple available physical magnitudes. In this regard, although the analysis of vibration signals represents the most significant source of information when the bearing fault diagnosis is addressed and faced [1,12], the proposed scheme allows the consideration of additional sources of information, that is, multiple vibration axis or even complementary physical magnitudes as the stator currents. Therefore, as Figure 3 depicts, the proposed approach may support a number of *N* available signals where the considered index *i*, for *I =* 1, 2,…, *N*, is used to identify each one of the considered physical magnitudes. Thus, regarding the proposed methodology, in the first stage, the available physical magnitudes are processed aiming to perform their characterization through the estimation of a potentially meaningful set of features. In this sense, the available signals, especially those related to the vibration of the electromechanical system in general, and the bearings in particular, are proposed to be processed into a *D* number of domains equal to three, that is, time domain (TD), frequency domain (FD) and time–frequency domain (TFD).

Although the proposed methodology adapts to any signal processing techniques to be considered in each domain, a reference set of features is proposed to be calculated from the available physical magnitudes as a trade-off between simplicity and performance. Additionally, as a general framework, the different signals that are available are first subjected to a segmentation procedure, where each acquired signal is individually divided into equal parts of one second. Thus, considering that $s_{i}$ refers to each available signal, for *i =* 1, 2, *…*, *N*, the segmentation procedure is performed by following Equation (8)

(8) $$s_{i}=\left[s_{i}^{1:L},\;s_{i}^{L+1:2L},\ldots ,\;s_{i}^{\left(\left(\left(n/L\right)-1\right)n\right)+1:n}\right]$$

where *L* refers to the length of the time windows, but, specifically, is the number of sampled points for each segment of the signal; *n* is the total number of sampled points for each available signal. Through this segmentation process, each available signal is divided in equal parts obtaining *n/L* segments. Dealing with periodic patterns in rotatory electromechanical machinery, this temporal duration assures enough statistical consistency in most of the practical applications (i.e., rotatory speeds higher than 500 rpm). However, the acquisition time can be increased without any loss of performance for those low-speed applications.

Thereby, each considered signal is processed by applying the TD analysis, specifically, a numerical set of *T =* 15 statistical time domain features is estimated from each segmented part of the available signals; as a result, a consecutive set of numerical features represented into a *T*-dimensional space is computed; precisely, a feature matrix composed by statistical time domain features is obtained, **T** ∈ ℝ^T^. On the other hand, for the FD analysis, the FFT technique is applied to each segmented part of the signals in order to obtain its representative frequency spectrum. Subsequently, a numerical set of 14 statistical features is calculated from each one of the obtained frequency spectrums. Additionally, six characteristic-bearing fault-related frequency components are estimated, that is, the first and second harmonic corresponding to the outer, inner and ball bearing faults. Therefore, in the FD analysis, a resulting set of *F =* 20 features are calculated from each frequency spectrum, and a representative *F*-dimensional frequency-domain feature matrix, **F** ∈ ℝ^F^, is calculated. Finally, for the TFD analysis, the acquired signals are, first, processed by means of one of the most suitable signal-decomposition techniques, the empirical mode decomposition (EMD), which allows obtaining a set of sub-signals containing the main oscillatory modes included in the original one. Thereby, the EMD technique is applied to each one of the segmented parts of the available signals and, following the related literature, the two first intrinsic modes functions (IMF), are taken into account for being characterized, since they contain the most significant information in terms of characteristic fault patterns. Later, the FFT technique is applied to each IMF to compute their corresponding frequency spectrum. The estimation of the same set of 14 statistical frequency-features is carried out for each IMF’s frequency spectrum. Therefore, for the TFD analysis, a set of *TF* = 28 features are estimated for each acquisition. As a result, a representative *TF*-dimensional time–frequency domain feature matrix, **TF** ∈ ℝ^T^^F^, is calculated.

Next, Table 1 and Table 2 summarize the proposed sets of statistical features estimated during the signal processing in the TD and FD analysis, respectively. These sets of statistical features have been included in other related works; its application has been preferred since they offer a high-performance signal characterization due to the capability of modeling trends and changes in the TD analysis, whereas, for the FD analysis, the estimation of these features allow to identify abrupt changes in the amplitude of those characteristic frequency components [34,35].

### 3.2. SAE Feature Learning

The second stage of the proposed method is focused on the self-adaptive extraction procedure to obtain a reduced set of features as a result of the learning procedure over the patterns described by the original feature matrices for each analyzed domain. This procedure is carried out by a multi-SAE structure that is applied over the feature matrices resulting from the analysis of the *N* available signals through a *D* number of domains of analysis, that is *D =* 3, **T*_i_***, **F*_i_***, and **TF*_i_***, for *i* = 1, 2,...*N*. Thus, it is proposed to consider as many SAE structures as available feature matrices to extract; for each matrix, a reduced set of features is considered while preserving most of the characteristic information that allows a proper reconstruction of the original signal. In this sense, it must be noted that performing a specific domain-based feature learning procedure over the extracted patterns leads to maximizing the characterization capabilities from each matrix. Indeed, the SAE seeks for an optimum codification in terms of posterior reconstruction error minimization, thus leading to the preservation of the underlying physical behavior of the signal from an unsupervised point of view.

However, as each matrix describes a different statistical distribution in its high-dimensional space, each SAE requires a specific hyperparameter tuning to reach a proper performance. Classically, in the related condition-monitoring literature, this procedure is faced empirically by means of a wrapper approach to maximize the final diagnosis performances. However, this approach leads to a high risk of overfitting, that is, the SAE is forced to prioritize those patterns providing higher diagnosis performances over those patterns that better characterize the original signal in terms of the reconstruction’s MSE. On the other hand, in most of the cases, several hyperparameter-tuning strategies have been classically proposed in order to optimize the hyperparameters regarding to a single model criterion, in which, the misclassification error is considered to obtain a model with a high performance. In this sense, this proposal faces the challenge of achieving the hyperparameter-tuning procedure by taking into account the trade-off between model accuracy (proper reconstruction of the original signals) and preservation of the characteristic information (underlying physical behavior of the signal). Therefore, the hyperparameter tuning for each considered SAE structure is faced through a heuristic search algorithm, the genetic algorithm (GA), where the fitness function of the GA is focused on the minimization of the MSE value during the reconstruction. In fact, the use of heuristic search algorithms, such as GA, has been widely used and preferred since the reached solutions are based on a stochastic optimization method. Moreover, one of the main benefits of using GA is that optimal or near-optimal solutions of a large-scale problem may be obtained under a reasonable computational time.

Therefore, in this proposal the hyperparameter optimization process is individually carried out by a GA for each considered SAE structure; the SAE hyperparameters that are optimized are (i) the coefficient for the L_2_ regularization term, (ii) the coefficient for the sparsity regularization term and (iii) the parameter for sparsity proportion. Thus, the optimization of the three mentioned hyperparameters is performed by following the next steps:

- **Step 1:** Initialization of the population: the chromosomes of the GA are initially defined with a logical vector containing three elements, where each element represents each one of the hyperparameters. Subsequently, a random initialization of the population is performed by assigning a specific value to each particular hyperparameter; in fact, the values assigned to each hyperparameter are within a predefined range of values. Once the initialization of the population is achieved, the procedure continues in step 2.
- **Step 2:** Evaluation of the population: in this step the fitness function is evaluated based on the minimization of the reconstruction error between the input and the output features. Specifically, the minimization of the reconstruction error is evaluated in terms of the MSE value following Equation (3), as mentioned in Section 2. Thereby, the optimization problem to be solved by the GA involves the search of those specific hyperparameter values that leads a high-performance feature mapping. Then, once the whole population is evaluated under a wide range of values, the condition of best hyperparameter values is analyzed and the procedure continues in step 4.
- **Step 3:** Mutation operation: the mutation of the GA produces a new population by means of the roulette wheel selection; the newly generated population takes into account the choice of the best fitness value achieved by the previously evaluated population. Moreover, a mutation operation that is based on the Gaussian distribution is applied during the generation of the new population. Subsequently, the procedure continues in step 2.
- **Step 4:** Stop criteria: there are two stop criteria for the GA: (i) the obtention of a reconstruction MSE value lower than a predefined threshold, 5%, and/or, (ii) reaching the maximum number of iterations, 1000. In the case of the first stop criterion, (i), the procedure is repeated iteratively until those optimal hyperparameter values are found until the GA evolves, then the procedure continues in step 3.

On the other hand, regarding the number of neurons for the hidden layers, multiple works reported in the related literature suggest a first hidden layer for expanding the number of neurons, and then the expansion is followed later with reduction in neurons in posterior layers [32]. In this regard, and following such good practices that have been validated in previous works, the SAEs are proposed to be constituted by a first hidden layer to expand neurons in a ratio from 5 to 10 times approximately, and three additional hidden layers reducing neurons in 1/3, 1/3 and 1/2 ratios approximately, thus resulting in a final hidden layer with a number of neurons equal to the number of considered classes, *C*. By means of this approach, each considered SAE structure provides the most representative *C* features that preserves the characteristic features of the signal for each considered domain as much as possible, that is, the most representative underlying physical behaviors seen from each domain that will be considered for posterior recognition. Thus, the SAE structures for TD, FD and TFD analysis are SAE*^T^_i_*, SAE*^F^_i_*, SAE*^TF^_i_*, respectively, SAE*^T^_i_*, SAE*^F^_i_*, SAE*^TF^_i_* ∈ ℝ*^C^*.

### 3.3. Data Fusion and Fault Diagnosis

Finally, a concatenated vector of *D* × *N* number of SAE structures, resulting from the consideration of *N* available signals that are processed into *N* domains per *C* considered classes (*D* × *N* × *C*), is considered as a feature fusion approach for classification purposes. Taking into consideration that all the posterior stages are focused on the learning of the most significant features from each available signal in all three domains, in this stage, the most significant patterns for their recognition are extracted. It should be noted that the proposed methodology allows a simple configuration for the classification task, since the input vector represents a processed set of features highlighting the representative characteristics of the signals.

In this regard, a simple softmax layer is proposed for diagnosis purposes. Thus, carrying out a one-neuron layer training based on a supervised approach. The proposed softmax layer provides the corresponding probability as part of the activation function values, resulting in a vector of *λ*_1_(*z*), *λ*_2_(*z*), *λ_C_*(*z*), where the *λ_j_*(*z*) represents the probability of *j*-th class. The calculation of *λ_j_*(*z*) is defined following:

(38) $$\lambda_{j}\left(z\right)=\exp\left(z_{j}\right)/\sum^{\;}\exp\left(z_{j}\right),\;j=1,2,\ldots ,C$$

(39) $$z_{j}=W_{j}M+b_{j}$$

## 4. Experimental Validation

### 4.1. Pulley-Belt Electromechanical System

**Figure 4:**
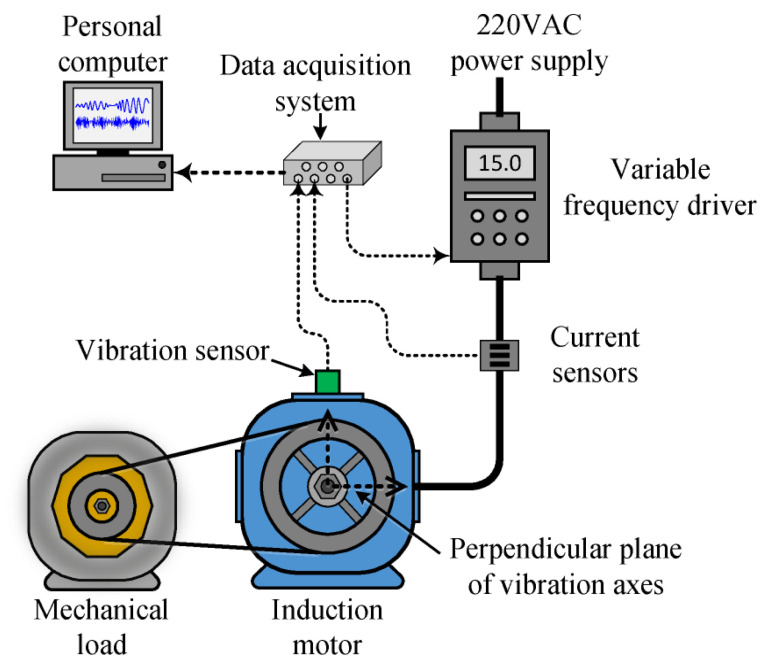
Flow chart of the laboratory electromechanical system based on a pulley-belt system and its wiring to perform the data acquisition.

The electromechanical system used to experiment with different bearing technologies is a self-designed laboratory test bench based on a pulley-belt system, as shown in Figure 4. The electromechanical system comprises a 971-W three-phase induction motor (IM), which model is WEG00136APE48T; the IM has one pair of poles and supports 220 VAC as a power supply. A variable frequency driver (VFD), model WEGCFW08, is used to feed the motor allowing the control of its rotational speed. Moreover, the motor is coupled by means of a pulley-belt system to an ordinary alternator. That produces a nominal load in the IM between 25% and 35%. The automotive alternator is used as a mechanical load and it produces a nominal load with variations within 25% to 35% in the IM. Such percentages of nominal loads are in terms of the stator current consumption in the IM and these values are estimated through experimental tests and by doing comparisons with the nominal current of the IM at its full load. On the other hand, although the alternator may be used as a power generation source to feed an arrangement of resistive loads, the performed experiments were carried out without considering the additional connection of resistive loads to the alternator. Thereby, the aforementioned percentages of load conditions are produced just by the rotational inertia of the alternator.

Regarding the acquisition of the data, two vibration axes and one stator current are measured and acquired with a proprietary data acquisition system (DAS) that is a low-cost design based on FPGA (field-programmable gate array). The designed DAS has a 12-bit 4-channel serial-output sampling analog-to-digital converter from Texas Instruments (i.e., model ADS7841). For the experimentation, different frequency values are set in the VFD to produce different rotational speeds in the IM and, for each operating frequency, the continuous measurement of the vibration signals and stator current is performed. Thus, the mechanical vibrations produced by the electromechanical system are acquired with an accelerometer model LIS3L02AS4 that is placed on the top of the IM, as Figure 4 illustrates; the acquired vibrations signals belong to the perpendicular plane of the rotating axis of the motor shaft. On the other hand, the stator current of one of the three phases of the IM is measured through a hall-effect sensor from Tamura Corporation model L08P050D15, such sensor has high linearity (i.e., 1%) and allows to perform measurements of 50 Amp; thus, the sensor is placed between the power supply lines, as shown in Figure 4. Furthermore, the used sensors are mounted individually on a PCB with its corresponding signal conditioning and anti-alias filtering. The sampling frequency to acquire the vibration signals and the stator current was set to 3000Hz and, for each performed experiment, 300 s of continuous operation are acquired and stored in a personal computer for posterior processing.

**Figure 5:**
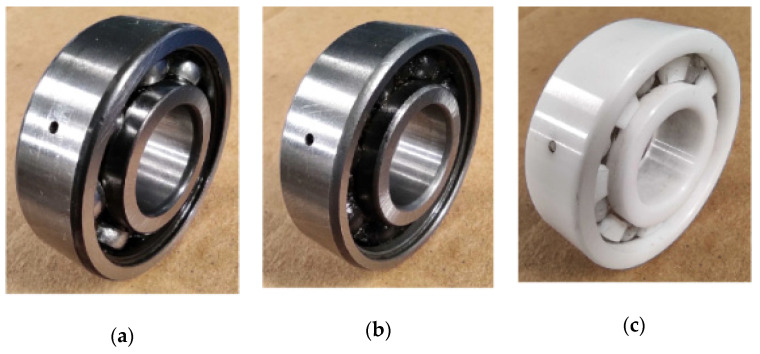
Set of damaged bearing used to be experimentally tested in the IM of an electromechanical system: (**a**) metallic damaged bearing, (**b**) hybrid damaged bearing and (**c**) full ceramic damaged bearing.

**Figure 6:**
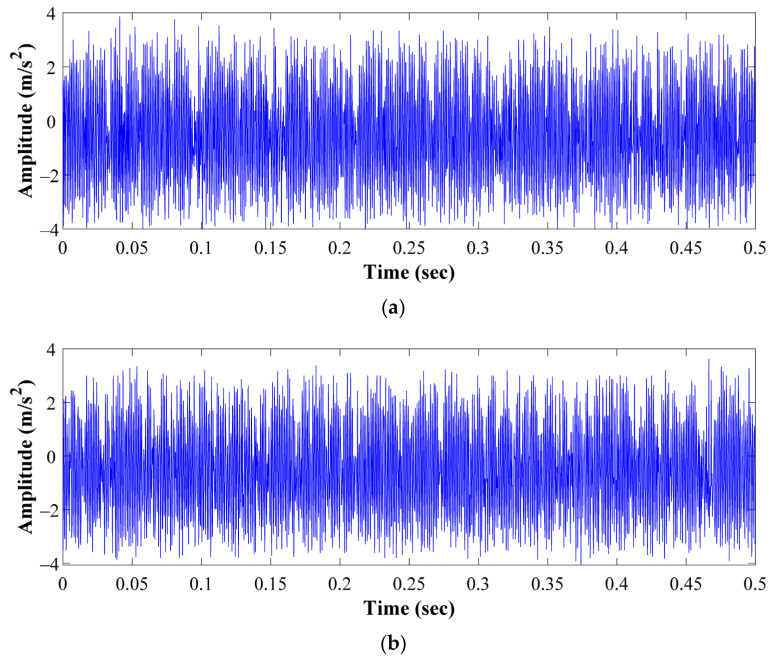

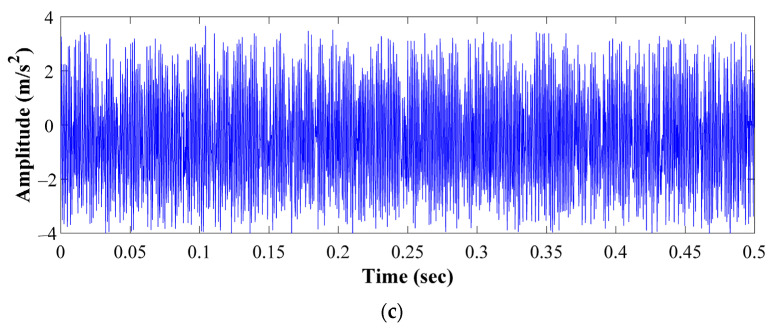
Vibration patterns acquired during the evaluation of the outer race faulty condition in: (**a**) the metallic bearing, (**b**) the hybrid bearing and (**c**) the full ceramic bearing.

**Figure 7:**
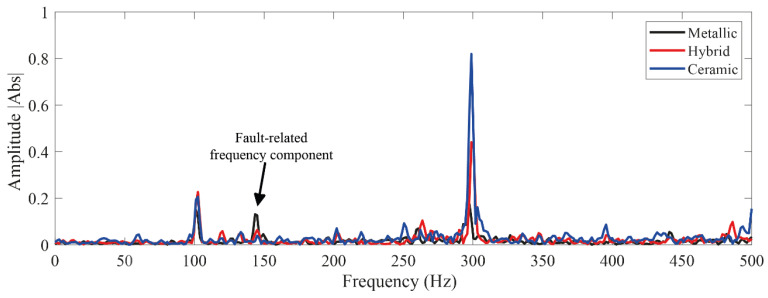
Frequency spectra obtained by applying the fast Fourier transform to the raw vibration signals of all considered bearing technologies: metallic, hybrid and full ceramic.

In regard to the assessed conditions, different bearing technologies are experimentally evaluated in the electrical motor to assess its corresponding condition. Hence, a metallic bearing, a hybrid bearing and a full ceramic bearing are experimentally tested. The bearing model is the 6203, and it is the end drive bearing of the IM. Two different bearing conditions are evaluated for each one of the considered technologies, that is, the healthy or normal condition (HC), and the outer race bearing defect or bearing damaged (BF) are tested. To produce the bearing defects, a metallic, a hybrid and a ceramic bearing were artificially damaged by drilling a hole with a tungsten drill bit of 1/16 of diameter; the drilled hole completely passed the bearing outer race of the three bearings. Thus, the drilled bearings are shown in Figure 5a–c for each corresponding technology: metallic, hybrid and full ceramic, respectively. Thus, for each considered bearing technology, both bearing conditions (HC and OD) are iteratively tested in the IM under different supply frequencies that are set in the VFD, that is, 5 Hz, 15 Hz, 50 Hz and 60 Hz. Thus, the condition assessment in different bearing technologies may represent a complex task, even more, whether a specific pattern is not shown. Theoretically, it is well-known that for bearings with defects in the inner race and/or outer race, the bearing system is under the influence of induced impacts when the balls get in contact with the defect area during the rotational working operation of the bearing. Although these impacts usually appear at intervals of time that are influenced by the size and shape of the defect, its appearance may not occur depending on the severity of the damage. In this sense, aiming to show the complexity of the addressed problem, from Figure 6a–c the vibration patterns acquired when the different bearing technologies are tested under the faulty condition, metallic, hybrid and full ceramic, are shown, respectively. As it can be appreciated, for any of the cases, a specific pattern does not exist that depicts the occurrence of the outer race faulty condition over the vibration patterns; specifically, the vibration patterns do not show periodic impacts that may be associated with the bearing defect. On the other hand, even though the appearing of periodic impacts on the vibration patterns may be induced by the influence of the outer race bearing fault in all considered bearing technologies, metallic, hybrid and full ceramic, respectively; the condition assessment and fault detection may be complicated due to the bearings under analysis have similar geometric properties. Additionally, aiming to provide a better understanding of the addressed problem, the corresponding frequency spectra for each raw vibration signal of Figure 6 are estimated by means of the fast Fourier transform. Thus, in Figure 7 is possible to observe the vibration spectra for all bearing technologies, metallic, hybrid and ceramic; as it can be appreciated, the theoretical fault-related frequency component of the outer race bearing defect is around 150 Hz when the bearings are operated at 50 Hz in the IM. Additionally, it should be highlighted that a clear difference between all estimated frequency spectra does not exist, that is, all the frequency spectra show similar amplitude values; therefore, additional signal processing techniques have to be implemented to improve the condition assessment.

### 4.2. Rolling Bearing CWRU Dataset

In order to verify the effectiveness of the proposed methodology, the public experimental dataset provided by the Case Western Reserve University Bearing Data Center (CWRU) has been also considered for being analyzed under the proposed DL-based fault diagnosis approach. Such database is considered as a standard database and it has been used widely used by many researchers to prove the effectiveness and applicability of their condition-monitoring approaches [30,36]; several condition-monitoring and fault diagnosis strategies have been specifically based on the analysis the CWRU data [15,16,17,30,31].

The test bench consists of a 1.49 kW reliance electric motor, a torque transducer, a dynamometer and an electronic controller; the database contains vibration data that have been collected from the experimental test rig when different damaged bearings are iteratively tested under four different loads conditions: 0, 0.74, 1.49 and 2.23 kW. Regarding the bearing conditions, besides the healthy condition or normal condition (HC), three different faulty conditions are introduced separately in the drive-end of the reliance electric motor, i.e., inner race fault (IF), outer race fault (OF) and ball fault (BF). The vibration signals were acquired through an accelerometer that was attached with a magnetic base at the drive-end of the reliance electric motor; the vibration data was collected with 12 kHz as a sampling frequency. Thereby, in order to validate the proposed diagnosis methodology in front of multiple bearing faults, the vibration signals corresponding to the HC, IF, OF and BF conditions with artificial single point faults of 0.014 in diameter are considered.

## 5. Results and Validation

### 5.1. Evaluation of the Diagnostic Model in the Pulley-Belt Electromechanical System

In a first validation stage, the proposed method is applied over the available signals acquired from the pulley-belt-based electromechanical system, that is, two vibration signals and one stator current. As aforementioned and for posterior notations, the considered indexes to identify these physical magnitudes are *i* = 1 and *i* = 2 for vibration and stator current, respectively. Thus, as described previously, the available signals were acquired during 300 s of the continuous operation of the IM and, according to the proposed method, each one of the acquired signals is segmented in equal parts of 1 s to generate a consecutive set of samples. As a result, three hundred individual segments were obtained from each considered signal by following Equation (8). Afterward, the multi-domain analysis was performed over each one of the segmented parts of the signals to achieve the signal characterization in the three mentioned domains, that is, DT, DF and DTF. In this regard, for the analysis carried out in TD, a set of 15 statistical features was computed from each segment of each available signal; therefore, for both vibration signals a feature matrix **T_1_** that contains 30 statistical features represented with 300 samples was achieved; meanwhile, the feature matrix achieved from the stator current **T_2_** consists of 15 statistical features with 300 samples. Subsequently, when the FD analysis is performed, from each segmented part of each available signal, the corresponding frequency spectrum is estimated by means of applying the FFT technique and then, from each resulting spectrum, the proposed set of 14 statistical features is calculated to characterize each spectrum into a set of representative numerical values. As a result, a characteristic feature matrix **F_1_** with 28 statistical features and 300 samples is carried out from both vibrations signals, while the characteristic matrix **F_2_** estimated from the stator current contains 14 statistical features with 300 samples. Additionally, for each available signal, six fault-related frequency features are taken into account; these additional features are the two first harmonic frequencies associated with three possible sources of fault in the bearing (i.e., outer, inner and ball bearing faults); thereby, these fault-related frequency features are represented by its corresponding amplitude resulting from each frequency spectrum. Accordingly, for the FD analysis, the resulting feature matrix **F_1_** of both vibration signals has a total of 40 features with 300 samples, and the feature matrix **F_2_** for the stator current has a total of 20 features with 300 samples. Then, during the TFD analysis, each segmented part is evaluated by the EMD technique in order to obtain the intrinsic modes associated with nonlinearities of the signals; thus, from each segmented part of the available signals, the two first intrinsic mode functions (i.e., *IMF*_1_ and *IMF*_2_) are considered as the two main intrinsic modes. Consecutively, the FFT technique is applied over each intrinsic mode and the resulting frequency spectra are then individually characterized by the estimation of the proposed set of 14 statistical features. Therefore, a feature matrix **TF_1_** with 56 statistical features and 300 samples is achieved for both vibration signals, whereas, for the stator current a feature matrix **TF_2_** with 28 features and 300 samples is obtained.

Thus, six representative feature matrices are estimated for each of the three experiments considering different bearing technologies. In summary, the feature matrices resulting from the analysis of the vibration signals in TD, FD and TFD are **T_1_**, **F_1_** and **TF_1_**, respectively; whereas, **T_2_**, **F_2_**, **TF_2_** are the resulting feature matrices from the analysis of the stator current in the TD, FD and TFD, respectively. Additionally, it should be clarified that these representative feature matrices are also estimated for each one of the supply frequencies considered (i.e., 5 Hz, 15 Hz, 50 Hz and 60 Hz). Following the proposed method, for each one of the bearing technologies and for each one of the available signals, the whole resulting feature matrices, considering all supply frequencies and taking into account the assessed bearing conditions, are grouped according to the domain of analysis. For example, in Table 3 the grouping of the feature matrices that are estimated from the vibrations signals is summarized; the feature matrices are grouped according to the domain of analysis, as it can be appreciated, and each corresponding grouping considers the bearing conditions (i.e., HC and BD conditions). These grouped matrices are represented by **T_1 *group*_**, **F_1 *group*_**, **TF_1 *group*_**, where Equation (40) gives the detail of a specific set of grouped matrices for **T_1 *group*_**. Thus, the same grouping is applied to feature matrices that are estimated from analyzing the stator current in the TD, FD and TFD for each one of the supply frequencies tested in the VFD and the obtained grouped matrices are **T_2 *group*_**, **F_2 *group*_** and **TF_2 *group*_**.

(40) $$T_{1\;group}=\left[\begin{array}{c} \begin{array}{c} \begin{array}{c} \underline{T_{1}@5\mathrm{Hz}@HC}\;\\ \underline{T_{1}@15\mathrm{Hz}@HC} \end{array}\\ \begin{array}{c} \underline{T_{1}@50\mathrm{Hz}@HC}\\ \underline{T_{1}@60\mathrm{Hz}@HC} \end{array} \end{array}\\ \begin{array}{c} \begin{array}{c} \underline{T_{1}@5\mathrm{Hz}@BD}\\ \underline{T_{1}@15\mathrm{Hz}@BD} \end{array}\\ \begin{array}{c} \underline{T_{1}@50\mathrm{Hz}@BD}\\ T_{1}@60\mathrm{Hz}@BD \end{array} \end{array} \end{array}\right]$$

Afterward, for each bearing technology that is tested under different supply frequencies, the corresponding feature matrices achieved from the multi-domain analysis of the available vibrations and stator current are grouped according to the domain of analysis (i.e., TD, FD and TFD), and then such matrices are individually used as the input space in several SAE structures. Indeed, a particular SAE structure for each one of the grouped sets of feature matrices for each considered bearing technology and available signal is defined. Thus, i.e., for the metallic bearing, the considered SAE structures for the grouped matrices of the vibration signal analyzed in TD, FD and TFD are ${\mathrm{SAE}}_{M}^{T_{1}}$, ${\mathrm{SAE}}_{M}^{F_{1}}$, ${\mathrm{SAE}}_{M}^{TF_{1}}$, respectively. Subsequently, as aforementioned, each SAE structure in conjunction with the GA is implemented over each corresponding feature space to be evaluated aiming to extract the most representative features that are related to the condition of the IM (i.e., the bearing condition). Moreover, as previously described, during the feature extraction procedure, the hyperparameters of the SAE network are automatically tuned by solving an optimization problem that uses the minimization of the reconstruction error as the fitness function. In this sense, in Table 4 and Table 5 the hyperparameters that are tuned by the GA for each SAE structure that is considered for each domain of analysis for the available signals, vibrations and stator current, are summarized, respectively. Additionally, Table 4 and Table 5 summarize the tuned hyperparameter for each of the bearing technologies tested. From the optimized hyperparameters for each feature space that are shown in Table 4 and Table 5, it should be noted that each hyperparameter shows variations between a specific range of values. That is, it can be assumed for the three feature domains and for the three bearing technologies, that all the SAE structures show a high-performance characterization of the input feature space by considering values in the *L*_2_ Regularization parameter about (2 ± 1) × 10^−5^, by considering values about (5 ± 3) × 10^−5^ in the Sparsity Regularization parameter and by considering the Sparsity Proportion with values about (0.5 ± 0.2).

**Figure 8:**
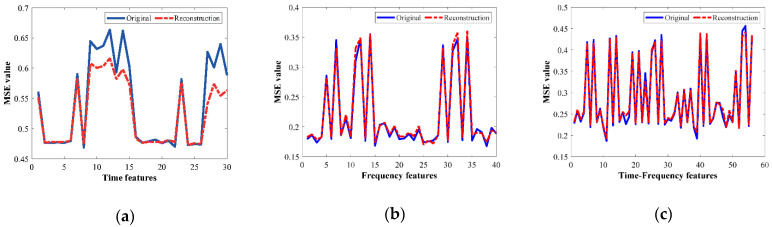
Qualitative representation of the original and reconstructed set of features for healthy metallic bearing at 5 Hz. (**a**) TD feature vibrations, (**b**) FD feature vibrations, (**c**) TFD feature vibrations.

On the other hand, Table 6 summarizes the corresponding MSE values that are carried out during the same optimization process are summarized. Indeed, such MSE values are considered as the metric to numerically compare the similarity between the original input features and the mapped and reconstructed features. As it can be noticed, all the MSE values summarized in Table 6 show small values which are desired to perform a high-performance characterization in the feature learning for the proposed SAE structures. Thus, to show the effectiveness of the SAE-based feature learning approach, from Figure 8a–c the original and reconstructed characteristic patterns that represent the HC in three different domains are shown, that is, TD, FD and TFD, respectively. These patterns belong to the metallic bearing tested with 5 Hz in the VFD, and as it is qualitatively appreciated, the reconstructed patterns match properly with the original ones.

**Figure 9:**
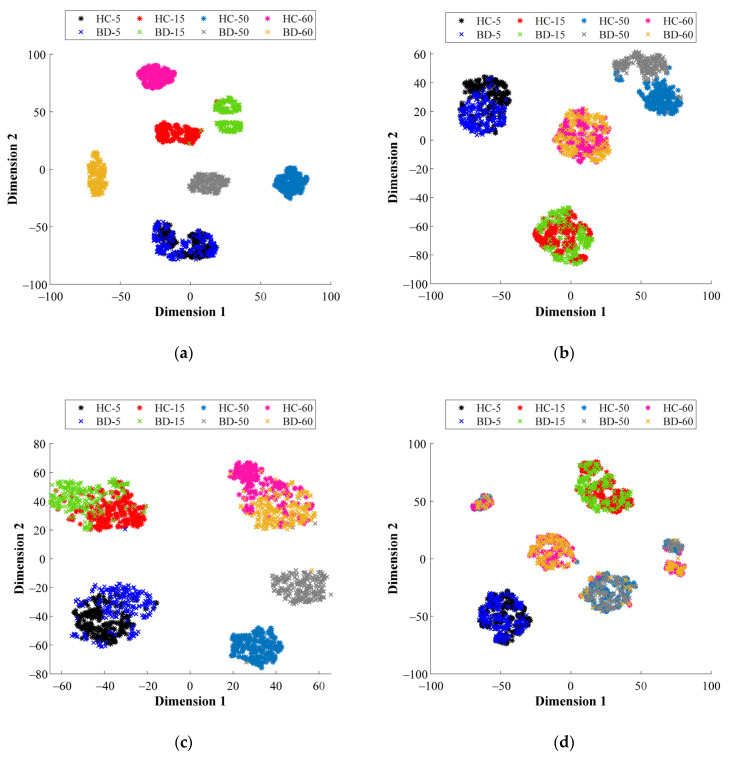
Two-dimensional visual representations of the data distribution achieved by the T-SNE technique over the resulting feature spaces mapped by their corresponding SAE of the metallic bearing when analyzing: (**a**) vibrations signals in FD, (**b**) stator current signature in FD, (**c**) vibrations signals in TFD and (**d**) stator current signature in TFD.

**Figure 10:**
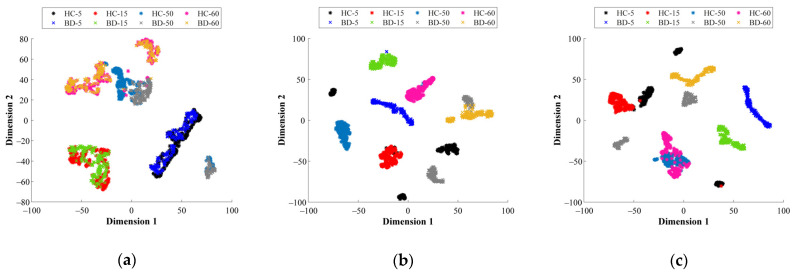
Achieved T-SNE representation of the data distribution into a 2D space of the resulting feature spaces mapped by their corresponding SAE when analyzing the stator current signature in the TD for each different bearing technology: (**a**) metallic, (**b**) hybrid and (**c**) full ceramic.

Aiming to highlight the contribution of the proposed multi-domain analysis in front of the characterization of physical magnitudes (i.e., vibrations and stator currents) for fault diagnosis applied to different bearing technologies, different visual representations of some of the feature spaces mapped by their corresponding SAE structure are shown next. Specifically, from Figure 9a–d the resulting feature spaces mapped by their corresponding SAE are projected into a 2D space through the T-SNE technique. Such representation allows interpretation of the data distribution of the considered bearing conditions that are tested at different operating frequencies. Thus, the 2D projections of Figure 9a,b represent both conditions, HC and BD, of the metallic bearing when the vibration signals and stator current are analyzed in the FD, respectively, while Figure 9c,d represent both conditions of the metallic bearing when the acquired signals are processed in the TFD, the vibrations and stator current, respectively. As it can be seen from Figure 9a–d, regardless of the considered domain of analysis, there exists an overlapping between the NC and the BD conditions for all the tested supply frequencies. Additionally, independently of the bearing technology, the consideration of a unique domain of analysis applied over a specific physical magnitude, i.e., the stator current, may not provide enough information that leads to a clear discrimination between bearing conditions. In this regard, from Figure 10a–c the visual representations of the data distribution are shown for the different bearing technologies, metallic, hybrid and ceramic, respectively. The achieved projections of Figure 10a–c represent both bearing conditions (HC and BD) when the stator current signature is analyzed in the TD, and as it is observed, an overlap appears between the considered bearing conditions.

**Figure 11:**
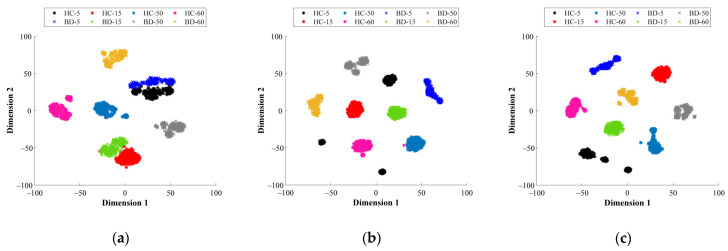
Resulting 2D representation of the data distribution of the mapped feature spaces by their corresponding SAE when all feature spaces domains are considered under the proposed fusion scheme (TD + FD + TFD) of all available signals (vibrations and stator current) are taken into account by the T-SNE for each different bearing technology: (**a**) metallic, (**b**) hybrid and (**c**) full ceramic.

Lastly, from Figure 10a–c the visual representations of the data distributions when all feature spaces domains are considered are shown, and as it can be noticed, regardless of the bearing technology and the considered supply frequency, the data fusion in multiple domains leads to a high-performance characterization of different bearing conditions. From Figure 11a–c, it can be observed that a clear separation is performed between the corresponding HC and BD conditions for the considered bearing technologies: metallic, hybrid and ceramic, respectively. Thus, condition-monitoring schemes based on data fusion through DL techniques represent a practical solution to achieve reliable condition assessments in multiple applications. Finally, aiming to provide the automatic fault diagnosis, the feature spaces mapped by their corresponding SAE structure are concatenated as a feature fusion approach, and then the concatenated vector is evaluated through a simple softmax layer that is proposed for diagnosis purposes. Thereby, in Table 7 the achieved percentages of classification accuracy for each considered bearing technology are summarized; additionally, in Table 7 the classification accuracies corresponding to the particular evaluation of each feature domain, TD, FD and TFD, and the proposed fusion scheme TD+FD+TFD, are included. As the obtained results depict, the proposed condition-monitoring scheme based on the multi-domain feature calculation and SAE feature learning leads to obtaining a high-performance classification accuracy.

Finally, aiming to highlight the effectiveness of the proposed method in front of classical condition-monitoring approaches that are based on machine learning, the feature matrices obtained by analyzing the available signals in TD, FD and TFD are subjected to a dimensionality reduction procedure by means of two well-known techniques, the PCA and the LDA. Afterward, the extracted features represented in a 2-dimensional space are evaluated through a classical NN-based classifier to achieve the condition assessment and fault diagnosis. Thus, two classical condition-monitoring structures are considered, PCA+NN and LDA+NN, to evaluate the different studied conditions for the considered bearing technologies. Both classical structures are individually applied to each particular feature domain, TD, FD and TFD, and are also applied under the fusion scheme TD + FD + TFD. The achieved classification ratios are summarized in Table 7. As it can be noticed, the classification ratios achieved by the proposed approach (SAE + Softmax) increase the classification performance up to 38.6% in the most critical case (TF features of the metallic bearing). Thus, the consideration of classical approaches, such as PCA + NN and LDA + NN, leads to obtaining low-performance classification ratios due to the fact that an additional technique to select those representative and meaningful features is required; meanwhile, the proposed approach results in high-performance classification ratios due to the ability of self-adapting to the extraction of the characteristic fault-related features from different signals that are processed in different domains.

Lastly, in order to highlight the advantages and effectiveness of the proposed SAE feature learning approach in front of classical approaches, a quantitative analysis of the performance metrics, accuracy, precision, recall and F1 score is performed. Thus, these performance metrics are estimated from the resulting classification matrices achieved by means of the simple softmax layer. Subsequently, in Table 8 the accuracy, precision, recall, and F1 score corresponding to each particular evaluation of each feature domain, TD, FD and TFD, and the proposed fusion scheme TD + FD + TFD, in the softmax layer are summarized. As it can be appreciated, all the performance metrics show values greater than 0.91 in the most critical cases, whereas the values of the performance metrics are greater than 0.97 in most cases. The averaged values of the performance metrics related to the confusion matrices achieved by classical approaches are 0.59, 0.74, 0.72 and 0.74 for the accuracy, precision, recall and F1 score, respectively, and 0.73, 0.83, 0.86 and 0.84 for the accuracy, precision, recall and F1, respectively, when the classical approaches PCA + NN and LDA + NN are evaluated, respectively. Therefore, the achieved results show that the proposed diagnosis methodology based on deep feature learning can be effectively applied to the diagnosis and identification of bearing faults for different bearing technologies, such as metallic, hybrid and ceramic bearings, in electromechanical systems. Additionally, the obtained results make the proposed approach feasible to be implemented as a part of the condition-based maintenance programs in industrial applications that involve rotating machinery.

### 5.2. Evaluation of the Diagnostic Model in the CWRU Database

In this section, the vibration signals of the CWRU bearing dataset are analyzed, aiming to evaluate the performance of the proposed DL-based diagnostic method in a reference framework, particularly in front of different metallic bearing failures. Thereby, the bearing conditions to be evaluated are the healthy or normal condition (HC), inner (IF), outer (OF) and ball (BF) faults with single-point faults of 0.014 in of diameter; such bearing conditions are also tested in combination with four different loads conditions: 0, 0.74, 1.49 and 2.23 kW. Subsequently, the vibration signals of the CWRU dataset are processed by following the practical application of the proposed DL-based diagnostic method and the collected vibration signals are first segmented in equal parts, resulting in a consecutive set of 150 samples, for each assessed bearing condition. Each sample of the consecutive sets of samples consists of 3000 data points, then, over each estimated sample the proposed multi-domain feature calculation is applied, that is, the processing of the vibration signals in TD, FD and TFD. The three resulting feature matrices are **T_1_**, **F_1_** and **TF_1_**, where each feature matrix comprises of 15, 20 and 28 features with 150 samples. Accordingly, for each one of the three resulting feature matrices, the total number of 150 samples corresponding to each tested condition is divided for training and testing purposes; in this sense, a random number of 100 samples are adopted for training and the remaining 50 samples for testing.

**Figure 12:**
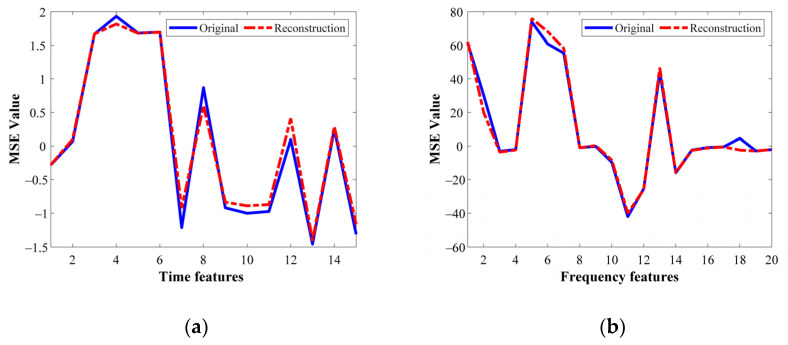

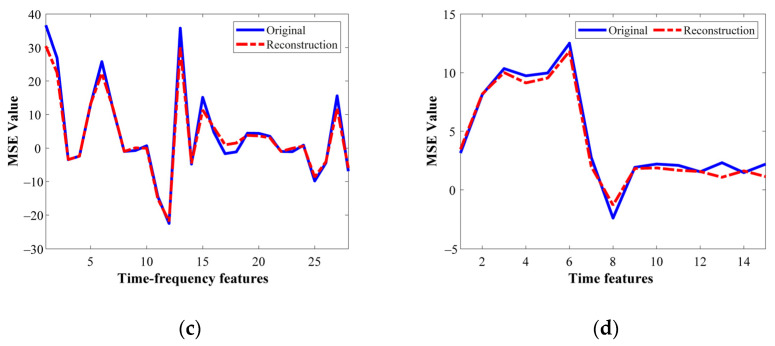
Comparison between the input feature patterns and its corresponding reconstruction obtained using the deep SAE-based feature extraction model on: (**a**) the NC represented in TD, (**b**) IF represented in TFD, (**c**) OF represented in TD and (**d**) OF represented in FD.

Afterward and similarly as in the previous experimental stage, the SAE feature learning is performed by a multi-SAE structure to be applied over the feature matrices resulting from the analysis of the unique available signal (*N* = 1) through the different three domains, that is, **T*_i_***, **F*_i_***, and **TF*_i_***, for *i* = 1,...*N*. Thus, the SAE structures for TD, FD and TFD analysis are SAE*^T^_i_*, SAE*^F^_i_* and SAE*^TF^_i_*, respectively. In this regard, the automatic optimization of the hyperparameters of the SAE network is carried out through the GA, aiming to minimize the MSE reconstruction value. Thereby, in Table 9 the achieved hyperparameters obtained through the application of the proposed tuning strategy are summarized; the average MSE value obtained during the optimization procedure shows values about (2 ± 1) × 10^−4^ that depict a high-performance characterization in the feature learning by the means of the SAE structure. Aiming to prove the effectiveness of the SAE-based feature learning approach, from Figure 12a–d the original and reconstructed feature patterns that represent assessed bearing conditions, NC, IF, OF and BF in different domains are shown, respectively. As it can be appreciated, the reconstructed patterns match properly with the original ones. Figure 12a shows the patterns corresponding to the HC represented in TD and their corresponding reconstruction obtained by the characterization of the SAE. As can be seen qualitatively, the representing feature patterns are correctly characterized, presenting an error of only about 5.07%. On the other hand, Figure 12b–d show the characteristic feature patterns of the vibration signals related to the IF, OF and BF bearing conditions represented in TFD, TD and FD, respectively. The reconstruction error achieved during the feature learning of the considered bearing fault conditions are: 1.99%, 6.08% and 2.43%, correspondingly.

**Figure 13:**
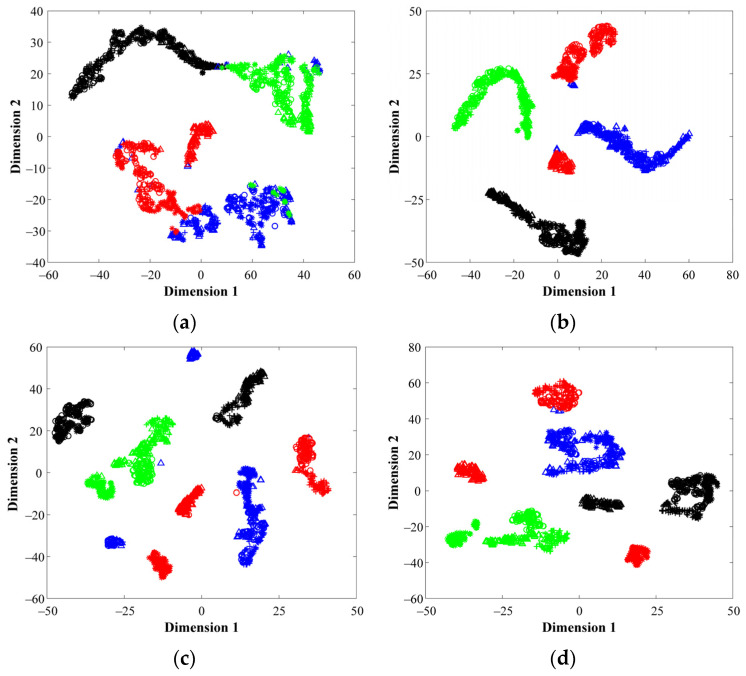
Feature visualization via t-SNE for: (**a**) time domain, (**b**) frequency domain (**c**) time–frequency domain and (**d**) fusion scheme. The colors used to represent the clusters shown in the resulting projections belong to the NC (●), BF (●), IF (●) and OF (●) conditions, where the different load conditions for each bearing condition are represented by the markers **△**, **+**, **○** and *, respectively.

Subsequently, the data distribution of the considered healthy states is projected into a 2D space by means of applying the T-SNE technique over the feature spaces mapped by their corresponding SAE structure. From Figure 13a, the representations of the considered bearing conditions, HC, IF, OF and BF are shown when the vibration signals of the CWRU dataset are analyzed in the TD, FD and TFD, respectively; Figure 13d shows the data distribution of the considered conditions when all feature space domains are taken into account, which is the proposed fusion approach (TD+FD+TFD). From these resulting projections, it should be mentioned that several samples appear overlapped when vibration signals are analyzed in TD (Figure 13a), whereas a better performance is achieved through the analysis in FD, TFD and the proposed fusion scheme (Figure 13b–d, respectively). As expected, the proposed DL-based diagnostic method exhibits the ability to learn and self-adapt the extraction of significant fault-related features from the available physical magnitudes for the characterization of electromechanical systems. Finally, the automatic fault diagnosis is performed through the proposed simple softmax layer, thus, the feature spaces mapped by its corresponding SAE structure are concatenated as a feature fusion approach. In Table 10, the achieved percentages of classification accuracy for each considered bearing condition of the CWRU dataset are summarized. Furthermore, in Table 10 the classification ratios corresponding to each particular feature domain, TD, FD and TFD, and the proposed fusion scheme, TD + FD + TDF, are appended. As the obtained results depict, the proposed fusion approach is superior to any of the individual domains due to capability of deep feature learning, which is supported by SAEs, for adapting and extracting the best fault-related characteristic patters of the electromechanical systems under evaluation. 

## 6. Conclusions

In this work is proposed a novel data-driven condition-monitoring methodology based on deep feature learning applied to fault diagnosis and identification in different bearings technology, that is, metallic, hybrid and full ceramic, in electromechanical systems. Advantageous results have been obtained through this proposed approach; indeed, there are three important aspects of this proposal that must be emphasized. The first one is that the use of a stacked autoencoder-neural network-based structure allows to perform a high-effective feature characterization during the assessment of bearing faults for different bearing technologies. Certainly, the important characteristic of the proposed SAE structure is its capability of learning and self-adapting to those meaningful features that are estimated from the available signal. The deep feature learning is supported by a proposed feature domain fusion approach that considers the signal processing in time, frequency and time–frequency domains through two processing techniques such as the FFT and the EMD. The second important aspect is the proposal and integration of the process that facilitates the tuning of the auto-encoder hyperparameters as part of the proposed approach; specifically, the use of a genetic algorithm in the tunning process leads to overcome the current limitations for practical application by industrial maintenance practitioners and leads to propose generalized-based approaches avoiding over-fitted solutions. Finally, the third aspect is related to the high-performance results obtained during the validation of the diagnosis method based on the deep feature learning approach in front of two different experimental scenarios, considering different bearing technologies and faults, different operating conditions, different electromechanical structures and even different acquisition systems. This fact proves the robustness and adaptability of the proposed method demonstrating that this proposal overcomes the challenge of noise immunity which represents a critical issue due to noise generation is inherent to the operation of rotating systems under industrial environments. Additionally, adaptability means other physical magnitudes and numerical features different from the considered in the proposal could be added since the method shows good feature learning capabilities during the characterization stage. Regarding the limitations of the method, the use and implementation of the genetic algorithm as a tool to search the optimal hyperparameter values may represent a challenge for industrial maintenance practitioners since prior knowledge is needed. The proposed DL diagnosis method is designed for the analysis of bearing faults, independently of the bearing technology; thus, this approach involves the validation of a common framework for the detection and classification of bearing faults that allows the consideration of multiple patterns. In this sense, future works consider the evolution towards evolving learning systems supported by the integration of novelty detection, incremental learning and transfer learning remain to be addressed, and that may allow the application of the deep feature learning method to identify the occurrence of faults in other elements such as gears, shafts, rotors and couplings, among others.

## Figures and Tables

**Table 1:** Proposed set of statistical time domain features estimated during the signal processing in the TD analysis, where each *y(j)* value belongs to each sample point of the processed signal for *j* = 1, 2, 3…*P*, with a total number *P* samples.

Statistical Feature	Mathematical Equation	
Mean	$t_{1}=\frac{1}{P}\;\overset{P}{\underset{j=1}{\sum }}\left|y_{j}\right|$	(9)
Maximum value	$t_{2}=max\left(x\right)$	(10)
Root mean square	$t_{3}=\sqrt{\frac{1}{P}\;\overset{P}{\underset{j=1}{\sum }}{\left(y_{j}\right)}^{2}}$	(11)
Square root mean	$t_{4}={\left(\frac{1}{P}\;\overset{P}{\underset{j=1}{\sum }}\sqrt{\left|y_{j}\right|}\right)}^{2}$	(12)
Standard deviation	$t_{5}=\sqrt{\frac{1}{P}\;\overset{P}{\underset{j=1}{\sum }}{\left(y_{j}-t_{1}\right)}^{2}}$	(13)
Variance	$t_{6}=\frac{1}{P}\;\overset{P}{\underset{j=1}{\sum }}{\left(y_{j}-t_{1}\right)}^{2}$	(14)
RMS Shape factor	$t_{7}=\frac{t_{3}}{\frac{1}{P}\;\sum_{j=1}^{P}\left|y_{j}\right|}$	(15)
SRM Shape factor	$t_{8}=\frac{t_{4}}{\frac{1}{P}\;\sum_{j=1}^{P}\left|y_{j}\right|}$	(16)
Crest factor	$t_{9}=\frac{t_{2}}{t_{3}}$	(17)
Latitude factor	$t_{10}=\frac{t_{2}}{t_{4}}$	(18)
Impulse factor	$t_{11}=\frac{t_{2}}{\frac{1}{P}\;\sum_{j=1}^{P}\left|y_{j}\right|}$	(19)
Skewness	$t_{12}=\frac{\sum^{\;}\left[{\left(y_{j}-t_{1}\right)}^{3}\right]}{t_{5}{}^{3}}$	(20)
Kurtosis	$t_{13}=\frac{\sum^{\;}\left[{\left(y_{j}-t_{1}\right)}^{4}\right]}{t_{5}{}^{4}}$	(21)
Fifth moment	$t_{14}=\frac{\sum^{\;}\left[{\left(y_{j}-t_{1}\right)}^{5}\right]}{t_{5}{}^{5}}$	(22)
Sixth moment	$t_{15}=\frac{\sum^{\;}\left[{\left(y_{j}-t_{1}\right)}^{6}\right]}{t_{5}{}^{6}}$	(23)

**Table 2:** Proposed set of statistical time domain features estimated from the frequency spectra during the signal processing in the FD analysis, where each *z(i)* is a spectrum for *i =* 1, 2,…,*Q*, and *Q* is the total number of lines for an estimated spectrum; *f_q_(i)* is the frequency value of the *i*-th spectrum line.

Statistical Feature	Mathematical Equation	
Mean	$f_{1}=\frac{1}{Q}\;\overset{Q}{\underset{i=1}{\sum }}z\left(i\right)$	(24)
Variance	$f_{2}=\frac{1}{Q-1}\;\overset{Q}{\underset{i=1}{\sum }}{\left(z\left(i\right)-f_{1}\right)}^{2}$	(25)
Third moment	$f_{3}=\frac{1}{Q{\left(\sqrt{f_{2}}\right)}^{3}}\;\overset{Q}{\underset{i=1}{\sum }}{\left(z\left(i\right)-f_{1}\right)}^{3}$	(26)
Fourth moment	$f_{4}=\frac{1}{Q{\left(\sqrt{f_{2}}\right)}^{2}}\;\overset{Q}{\underset{i=1}{\sum }}{\left(z\left(i\right)-f_{1}\right)}^{4}$	(27)
Grand mean	$f_{5}=\frac{\sum_{i=1}^{Q}f_{q}\left(i\right)\;z\left(i\right)}{\sum_{i=1}^{Q}z\left(i\right)}$	(28)
Standard deviation 1	$f_{6}=\sqrt{\frac{\sum_{i=1}^{Q}{\left(f_{q}\left(i\right)-f_{5}\right)}^{2}z\left(i\right)}{Q}}$	(29)
C Factor	$f_{7}=\sqrt{\frac{\sum_{i=1}^{Q}f_{q}{\left(i\right)}^{2}\;z\left(i\right)}{\sum_{i=1}^{Q}z\left(i\right)}}$	(30)
D Factor	$f_{8}=\sqrt{\frac{\sum_{i=1}^{Q}f_{q}{\left(i\right)}^{4}\;z\left(i\right)}{\sum_{i=1}^{Q}f_{q}{\left(i\right)}^{2}\;z\left(i\right)}}$	(31)
E Factor	$f_{9}=\frac{\sum_{i=1}^{Q}f_{q}{\left(i\right)}^{2}\;z\left(i\right)}{\sqrt{\sum_{i=1}^{Q}z\left(i\right)\;\sum_{i=1}^{Q}f_{q}{\left(i\right)}^{4}\;z\left(i\right)}}$	(32)
G Factor	$f_{10}=\frac{f_{6}}{f_{5}}$	(33)
Third moment 1	$f_{11}=\frac{\sum_{i=1}^{Q}{\left(f_{q}-f_{5}\right)}^{3}\;z\left(i\right)}{Q\;f_{6}^{3}}$	(34)
Fourth moment 1	$f_{12}=\frac{\sum_{i=1}^{Q}{\left(f_{q}\left(i\right)-f_{5}\right)}^{4}\;z\left(i\right)}{Q\;f_{6}^{4}}$	(35)
H Factor	$f_{13}=\frac{\sum_{i=1}^{Q}{\left(f_{q}\left(i\right)-f_{5}\right)}^{1/2}\;z\left(i\right)}{Q\;\sqrt{f_{6}}}$	(36)
J Factor	$f_{14}=\frac{\left(f_{7}+f_{8}\right)}{f_{1}}$	(37)

**Table 3:** Representation of the grouping of the feature matrices computed by means of analyzing the vibration signals in TD, FD and TFD for each supply frequency for the considered bearing conditions; where the notations *HC* and *BD* represent the healthy condition and the bearing damaged condition.

		Feature Domain		
		Time	Frequency	Time–Frequency
**Related condition**	**Normal**	**T_1_** @5Hz@*HC*	**F_1_** @5Hz@*HC*	**TF_1_** @5Hz@*HC*
		**T_1_** @15Hz@*HC*	**F_1_** @15Hz@*HC*	**TF_1_** @15Hz@*HC*
		**T_1_** @50Hz@*HC*	**F_1_** @50Hz@*HC*	**TF_1_** @50Hz@*HC*
		**T_1_** @60Hz@*HC*	**F_1_** @60Hz@*HC*	**TF_1_** @60Hz@*HC*
	**Damaged**	**T_1_** @5Hz@*BD*	**F_1_** @5Hz@*BD*	**TF_1_** @5Hz@*BD*
		**T_1_** @15Hz@*BD*	**F_1_** @15Hz@*BD*	**TF_1_** @15Hz@*BD*
		**T_1_** @50Hz@*BD*	**F_1_** @50Hz@*BD*	**TF_1_** @50Hz@*BD*
		**T_1_** @60Hz@*BD*	**F_1_** @60Hz@*BD*	**TF_1_** @60Hz@*BD*
**Grouped matrices**		**T_1 *group*_**	**F_1 *group*_**	**TF_1 *group*_**

**Table 4:** Parameters tuned by the GA for each SAE structure that is considered for each domain of analysis for the vibration signals used in the stacked AE; all technology materials.

Material	Feature Domain	Hyperparameters		
		*L*_2_ Regularization	Sparsity Regularizatio	Sparsity Proportion
Metallic	Time (T_1 *group*_)	1.31 × 10^−5^	7.384 × 10^−5^	0.664
	Frequency (F_1 *group*_)	1.616 × 10^−5^	5.761 × 10^−5^	0.655
	Time–Frequency (TF_1 *group*_)	3.250 × 10^−5^	7.164 × 10^−5^	0.779
Ceramic	Time (T_1 *group*_)	2.090 × 10^−5^	8.037 × 10^−5^	0.132
	Frequency (F_1 *group*_)	2.879 × 10^−5^	1.407 × 10^−5^	0.479
	Time–Frequency (TF_1 *group*_)	2.213 × 10^−5^	5.277 × 10^−5^	0.854
Hybrid	Time (T_1 *group*_)	2.212 × 10^−5^	7.785 × 10^−5^	0.176
	Frequency (F_1 *group*_)	1.276 × 10^−5^	2.406 × 10^−5^	0.560
	Time–Frequency (TF_1 *group*_)	1.064 × 10^−5^	4.435 × 10^−5^	0.240

**Table 5:** Parameters tuned by the GA for each SAE structure that is considered for each domain of analysis for the stator current signature used in the stacked AE; all technology materials.

Material	Feature Domain	Hyperparameters		
		*L*_2_ Regularization	Sparsity Regularizatio	Sparsity Proportion
Metallic	Time (T_2 *group*_)	2.277 × 10^−5^	7.108 × 10^−5^	0.3228
	Frequency (F_2 *group*_)	1.077 × 10^−5^	8.433 × 10^−5^	0.2026
	Time–Frequency (TF_2 *group*_)	2.277 × 10^−5^	7.108 × 10^−5^	0.3228
Ceramic	Time (T_2 *group*_)	1.675 × 10^−5^	6.944 × 10^−5^	0.4743
	Frequency (F_2 *group*_)	1.320 × 10^−5^	4.897 × 10^−5^	0.546
	Time–Frequency (TF_2 *group*_)	1.821 × 10^−5^	4.950 × 10^−5^	0.678
Hybrid	Time (T_2 *group*_)	2.228 × 10^−5^	2.824 × 10^−5^	0.332
	Frequency (F_2 *group*_)	1.625 × 10^−5^	4.365 × 10^−5^	0.391
	Time–Frequency (TF_2 *group*_)	3.384 × 10^−5^	7.621 × 10^−5^	0.4227

**Table 6:** MSE error achieved by the GA during the tunning and optimization of hyperparameters of the considered SAE structures for each domain of analysis, all technology materials.

Material	Feature Domain	MSE Error	
		Vibrations (*i* = 1)	Current (*i* = 2)
Metallic	Time (T*_i group_*)	1.071 × 10^−4^	3.689 × 10^−4^
	Frequency (F*_i group_*)	3.113 × 10^−4^	4.451 × 10^−4^
	Time–Frequency (TF*_i group_*)	4.348 × 10^−4^	1.137 × 10^−4^
Ceramic	Time (T*_i group_*)	7.8202 × 10^−5^	1.990 × 10^−4^
	Frequency (F*_i group_*)	1.894 × 10^−4^	2.786 × 10^−4^
	Time–Frequency (TF*_i group_*)	1.794 × 10^−4^	1.464 × 10^−4^
Hybrid	Time (T*_i group_*)	6.550 × 10^−5^	1.995 × 10^−4^
	Frequency (F*_i group_*)	3.543 × 10^−4^	2.778 × 10^−4^
	Time–Frequency (TF*_i group_*)	1.230 × 10^−4^	4.882 × 10^−4^

**Table 7:** Achieved classification ratios through the SoftMax layer for the particular evaluation of each feature domain and the proposed fusion scheme.

Material	Feature Domain	Proposed Method		PCA + NN		LDA + NN	
		Training	Test	Training	Test	Training	Test
Metallic	Time (T_1,2 *group*_)	99.1%	99.3%	60.5%	62.1%	73.3%	73.5%
	Frequency (F_1,2 *group*_)	96.5%	95.8%	68.6%	70.7%	78.3%	79.9%
	Time–Frequency (TF_1,2 *group*_)	95.3%	96%	78.9%	80.8%	71.5%	65.8%
	Fusion scheme (T_1,2 *group*_ + F_1,2 *group*_ + TF_1,2 *group*_)	100%	100%	81.2%	83.1%	62.5%	62.7%
Ceramic	Time (T_1,2 *group*_)	97%	96.5%	69.5%	70.8%	69.0%	71.9%
	Frequency (F_1,2 *group*_)	100%	100%	72.8%	73.9%	86.9%	88.6%
	Time–Frequency (TF_1,2 *group*_)	98.8%	98.8%	84.7%	88.8%	87.5%	87.9%
	Fusion scheme (T_1,2 *group*_ + F_1,2 *group*_ + TF_1,2 *group*_)	100%	100%	73.0%	73.2%	75.0%	75.0%
Hybrid	Time (T_1,2 *group*_)	99.8%	99.3%	81.0%	81.9%	87.4%	87.5%
	Frequency (F_1,2 *group*_)	100%	100%	84.2%	84.4%	87.9%	88.0%
	Time–Frequency (TF_1,2 *group*_)	99.9	99.8%	81.1%	86.7%	82.8%	84.0%
	Fusion scheme (T_1,2 *group*_ + F_1,2 *group*_ + TF_1,2 *group*_)	100%	100%	75.2%	75.3%	70.3%	71.4%

**Table 8:** Performance metrics of the proposed method based on SAE and SoftMax layer for the particular evaluation of each feature domain and the proposed fusion scheme.

Material		Accuracy		Precision		Recall		F1 Score	
		Training	Test	Training	Test	Training	Test	Training	Test
**Metallic**	**Time**	0.98	0.97	0.99	0.98	0.99	0.97	0.99	0.97
	**Frequency**	0.93	0.91	0.98	0.98	0.94	0.92	0.95	0.94
	**Time–Frequency**	0.95	0.96	0.97	0.98	0.97	0.97	0.97	0.97
	**Fusion scheme**	1.00	1.00	1.00	1.00	1.00	1.00	1.00	1.00
**Ceramic**	**Time**	0.96	0.96	0.98	0.98	0.98	0.97	0.98	0.97
	**Frequency**	1	1	1	1	1	1	1	1
	**Time–Frequency**	0.99	0.98	0.99	0.98	0.99	0.99	0.99	0.98
	**Fusion scheme**	1	1	1	1	1	1	1	1
**Hybrid**	**Time**	0.99	0.99	0.99	0.99	0.99	1	0.99	0.99
	**Frequency**	1	1	1	1	1	1	1	1
	**Time–Frequency**	0.99	0.99	0.99	1	0.99	0.99	0.99	0.99
	**Fusion scheme**	1	1	1	1	1	1	1	1

**Table 9:** Hyperparameter used in a deep SAE-based model for vibration signals in CWRU database.

Feature Domain	Hyperparameters		
	*L*_2_ Regularization	Sparsity Regularization	Sparsity Proportion
Time (T_1_)	8.0 × 10^−5^	8.0 × 10^−5^	0.55
Frequency (F_1_)	8.0 × 10^−5^	8.0 × 10^−5^	0.45
Time–Frequency (TF_1_)	8.0 × 10^−5^	8.0 × 10^−5^	0.50

**Table 10:** Resulting classification ratios through the SoftMax layer for the particular evaluation of each feature domain and the proposed fusion scheme.

Feature Domain	Average Accuracy	
	Training	Test
Time	96.2%	94.5%
Frequency	99.8%	98.5%
Time–Frequency	98.4%	96.8%
Fusion Scheme	100.0%	99.8%

## Data Availability

The CRWU dataset may found at: https://csegroups.case.edu/bearingdatacenter/pages/download-data-file (accessed on 25 August 2021).
